# Prevalence and Antimicrobial Resistance Profile of *Salmonella* Isolated from Human, Animal and Environment Samples in South Asia: A 10-Year Meta-analysis

**DOI:** 10.1007/s44197-023-00160-x

**Published:** 2023-10-26

**Authors:** Himel Talukder, Shamsul Alam Roky, Konad Debnath, Binayok Sharma, Juned Ahmed, Sawrab Roy

**Affiliations:** 1 Department of Epidemiology and Public Health, Sylhet Agricultural University, Sylhet, Bangladesh; 2 Department of Geography and Environmental Sustainability, University of Oklahoma, Norman, OK USA; 3 Department of Dairy Science, Sylhet Agricultural University, Sylhet, Bangladesh; 4 International Centre for Diarrheal Disease Research, Dhaka, Bangladesh; 5 Department of Medicine, Sylhet Agricultural University, Sylhet, Bangladesh; 6 Department of Animal Science, Purdue University, West Lafayette, IN USA; 7 Department of Pathology, Sylhet Agricultural University, Sylhet, Bangladesh; 8 School of Veterinary Medicine, Texas Tech University, Amarillo, TX 79106 USA; 9 Department of Microbiology and Immunology, Sylhet Agricultural University, Sylhet, Bangladesh; 10 Department of Veterinary Pathobiology, College of Veterinary Medicine, University of Missouri, Columbia, MO USA

**Keywords:** Prevalence, Antimicrobial resistance, *Salmonella*, South Asia

## Abstract

*Salmonella* is a foodborne zoonotic bacterium, and the antimicrobial-resistant strains of *Salmonella* are a worldwide health concern. Herein, we employed a meta-analysis to determine the pooled prevalence of *Salmonella* and its antimicrobial resistance status in human, animal, and environmental isolates in South Asia. To this end, we followed the standard guideline of Preferred Reporting Items for Systematic Reviews and Meta-Analyses (PRISMA) statements for searching literature in three databases namely PubMed, Google Scholar, and CAB abstracts, and a total of 100 eligible datasets were finally included which were published from January 2010 to June 2021. In the pooled prevalence of *Salmonella* in South Asia, the random model effect was 14.47% (95% CI: 10.17–20.19) with a high degree of heterogeneity (*I*^2^, 99.8%) and overall antimicrobial resistance was 70% (95% CI: 63.0–76.0) with a heterogeneity of 23.6%. The temporal distribution of the overall antimicrobial resistance (%) against *Salmonella* was increased from 53 to 77% within 10 years. Out of 18 distinct *Salmonella* serotypes, *S. enterica* was highly prevalent (14.22%, 95% CI: 4.02–39.64) followed by *S.* *pullorum* (13.50%, 95% CI: 5.64–29.93) with antimicrobial resistance (%) were 86.26 and 90.06, respectively. Noteworthy, nalidixic acid (74.25%) and tetracycline (37.64%) were found mostly resistant to *Salmonella* whereas ceftriaxone (1.07%) and cefixime (1.24%) were sensitive. This systematic review demonstrated that overall antibiotic resistance profiles of *Salmonella* are increasing over time in South Asia. Thus, adequate hygienic practices, proper use of antimicrobials, and implementation of antibiotic stewardship are imperative for halting the *Salmonella* spread and its antimicrobial resistance.

**Supplementary Information:**

The online version contains supplementary material available at 10.1007/s44197-023-00160-x.

## Introduction

*Salmonella* is a Gram-negative, rod-shaped, non-spore-forming, and facultative anaerobic bacterium belonging to the Enterobacteriaceae family that can produce diseases in humans and animals [1]. Under the genus *Salmonella*, there are two species, namely *S. bongori* and *S. enterica.* Although *S. bongori* is primarily found in cold-blooded animals, this species also can infect humans [2]. On the other hand, *Salmonella enterica* is widely abundant and comprises over 2600 serovars which are categorized into typhoidal and non-typhoidal groups. Although these two groups have a resemblance genetically, they can produce various diseases with distinct immune responses [3]. Among all non-typhoidal *Salmonella* (NTS) serovars globally, around 50% of human isolates were found from *S. enterica* serovar Typhimurium and *S. enterica* serovar Enteritidis [4].

*S. enterica* causes a wide range of food and water-borne diseases both in humans and animals [5]. In humans, approximately 93.8 million cases of foodborne illness and/or gastroenteritis and 155,000 death cases are reported yearly due to non-typhoidal *Salmonella* [6, 7]. The global prevalence of *Salmonella* is high, and non-typhoidal *Salmonella* infections are reported across the world. However, the prevalence of *Salmonella* varies from region to region [8]. For instance, in the USA, NTS has been reported as the second-most causal bacteria for foodborne illness [9] whereas typhoidal salmonellosis is highly prevalent in South and South-East Asia. Again, invasive non-typhoidal *Salmonella* has been recorded to cause bacteremia with high morbidity in the sub-Saharan African region [8].

*Salmonella* has a wide host range such as reptiles, avian species, and mammals including humans [10]. NTS is usually transmitted to humans by contaminated foods, animals, animal products, and manures [11, 12]. Some studies found an association between *Salmonella*-contaminated fruits and vegetables with food poisoning. Pathogenicity studies showed that *Salmonella* has some unique properties to cross a larger number of barriers and invade different cells [5]. NTS is often reported as an important foodborne pathogen causing gastrointestinal disorders, different localized infections, and bacteremia. These bacteria can develop worse conditions in immunosuppressive humans [13], especially malaria-infected patients, malnourished children, and human immunodeficiency virus (HIV) patients [4]. Notwithstanding treatment and preventive strategies being implemented, millions of new typhoid infections are being reported globally every year [14]. Interestingly, *S. typhimurium* not only infects humans and animals but also can use plants as their alternative hosts. Infections in humans, animals, and plants by *Salmonella* have raised questions about their host specificity [15].

*Salmonella* is one of the most reported zoonotic pathogens, and the antimicrobial-resistant (AMR) strains of *Salmonella* are a big concern for public health [16]. Even though NTS commonly causes gastrointestinal infections worldwide, most of the strains cause mild gastroenteritis which is usually not required to treat with antibiotics [17]. However, there are some factors that help *Salmonella* to be more pathogenic and a threat to public health. For instance, genetic modification and genomic evolution in *Salmonella* have increased virulence and have made them resistant to multiple drugs [17]. Antibiotic-induced selective pressures cause mutations in chromosomal genes and plasmid leading to continuous genetic evolution in *Salmonella*. Again, horizontal gene transfer may also contribute to the spread of AMR genes. The acquisition and spread of resistant genes are significantly affected by the exchange between plasmid(s) and the bacterial chromosome as well as the integration of resistant genes into specialized genetic components known as integrons [17, 18]. In fact, poultry that has never been raised with antibiotics has meat contaminated with antibiotic-resistant *Salmonella* [9]. Moreover, wastewater in hospitals is a hotspot for AMR pathogens, and hospitals are contributing to the spread of resistant pathogens [19]. Overall, there are several underlying factors for developing AMR, and the root causes are complex, particularly in developing countries. Inappropriate use of antimicrobials and lack of knowledge about antibiotics usages are crucial factors. The use of antimicrobials in animals and plants is also generating resistant bacteria [20]. AMR serotypes of *Salmonella* can also be transmitted with wild birds such as vultures [16]. AMR is generating serious challenges for health and the economy. It is estimated that ten million deaths may occur by 2050 due to AMR. The World Bank has also estimated that by 2050, the global GDP may fall by 1.1–3.8% due to the independent impacts of AMR [21].

South Asia is at high risk in terms of the emergence and spread of antimicrobial resistance [22]. Despite the increasing knowledge of the prevalence of *Salmonella* and its AMR profile which is mostly reported by individual and local surveillance study(s), comprehensive and robust study of the prevalence and AMR pattern in South Asia is poorly characterized. Thus, this meta-analysis includes a comprehensive evaluation of scientific literature published between January 2010 to June 2021 on antimicrobial resistance by species-specific *Salmonella* serotypes isolated from the environment, animals, and humans in South Asia.

## Methodology

### Study Design and Systematic Review Protocol

The outcome of interest in this study includes any species under the genus of *Salmonella* isolated from humans or animals or environment using cultural, immunological, or molecular diagnostic methods, and AMR patterns of *Salmonella*. The outcome measure is the prevalence of *Salmonella* based on the peer-reviewed publication which may contain one or more datasets, depending on different sampling techniques, sources, time, and locality. We used three different databases for searching literature: PubMed (https://pubmed.ncbi.nlm.nih.gov/), Google Scholar (https://scholar.google.com/), and CAB abstracts (https://www.cabi.org/publishing-products/cab-abstracts/). The search key was *Prevalence OR Incidence OR Occurrence AND Salmonella OR Salmonellosis AND antimicrobial resistance OR antibiotic resistance AND (Bangladesh/India/Pakistan/Nepal/Bhutan/Maldives/Sri Lanka/Afghanistan).* Articles published from January 2010 to June 2021 were included in this study. The last search was conducted on May 24, 2021. This meta-analysis was conducted by following the standard guideline of Preferred Reporting Items for Systematic Reviews and Meta-Analyses (PRISMA) statements [23].

### Selection Criteria

The inclusion criteria for all the eligible studies included in the meta-analysis were: articles published primarily on the quantitative prevalence of *Salmonella* spp. in humans, animals, and environment in South Asia (Bangladesh, India, Pakistan, Nepal, Bhutan, Maldives, Sri Lanka, and Afghanistan); the study included descriptive (surveys) or observational (cross-sectional, case–control and cohort) and not clinical trials; and articles only reported in English on antibiotic resistance of *Salmonella* published between January 2010 to June 2021.

Studies excluded from this meta-analysis were book and book chapters, review papers, unpublished studies, proceedings, and theoretical models. Studies were further excluded if the diagnostic test was not mentioned and had overlapping data with another included study.

### Data Extraction

Based on the ‘*search keywords*’, the titles and abstracts were initially examined, and full-text articles were downloaded to determine the eligibility. Primary information including author(s) name, year of publication, location, total sample size, number of positive samples, species of *Salmonella*, and its antibiotic resistance profile were collected individually from the publication and entered into Microsoft Excel. Two authors independently reviewed and evaluated the full texts for the eligibility of final inclusion. In our study, we examined a large dataset of 822,120 isolates. We found a total of 28,810 isolates that tested positive for *Salmonella*. It is important to note that we were able to specifically identify 18 distinct *Salmonella* serotypes out of these positive cases, whereas the remaining cases were non-specific in terms of serotype identification. In these studies, different types of samples were collected such as blood, feces, gut, and anal swab from animals; food, insect, soil, and water from the environment; blood, serum, stool, and hand swab from humans (Supplementary Table 2). Diagnostic approaches used for these studies were culture, biochemical, PCR, and serological. All antimicrobial susceptibility tests (AST) were done by using disc diffusion method; therefore, no subgroup analysis was performed on AST.

### Statistical Analysis

A random-effect meta-analysis was carried out to estimate the prevalence of *Salmonella* with 95% Confidence Interval (CI) in South Asia. Between-study variations were identified by using Cochran’s *Q* test to measure the heterogeneity which indicates whether the variation in the studies is more than the expected level by chance (*P* < 0.05 were considered significant heterogeneity). Higgins’s *I*^2^ value was used to determine the percentage of total variance in effect estimates among the studies which was attributable to heterogeneity rather than chance. *I*^2^ values of more than 50% were considered as high heterogeneity [24]. Publication bias may affect the pooled effect estimation. Therefore, we also performed Egger’s test and funnel plot to explore the potential publication bias. Antimicrobial resistance percentage was defined as the number of antimicrobials were found resistant divided by total number of antimicrobials were tested. A subgroup meta-analysis was conducted on different subsets of data to find out the prevalence of *Salmonella* in different sources, locations, countries, and time-periods. The test for subgroup differences was based on Cochran’s *Q* value which indicates the presence of heterogeneity among subgroups. The prevalence estimates of different datasets were pooled using the DerSaimonian-Laird random-effect method [25]. Additionally, meta-regression analysis was used to further investigate the heterogeneity in different subsets of the group. Four possible groups were examined: Country, Source (Human, Animal, or Environment), Location (Rural, Urban, Both), and Time (2010–2013, 2014–2017, 2018–2021). Initially, a univariate meta-regression model was employed to determine the association between different groups and the prevalence of *Salmonella*. Groups with *P* < 0.2 in univariate analysis were included in the final multivariate model. All the analyses were performed using the ‘*Meta*’, ‘*Metafor*’, and ‘*DmetaR*’ packages of the open-source R (version 4.0.3).

## Results

### Study Selection

**Fig. 1:**
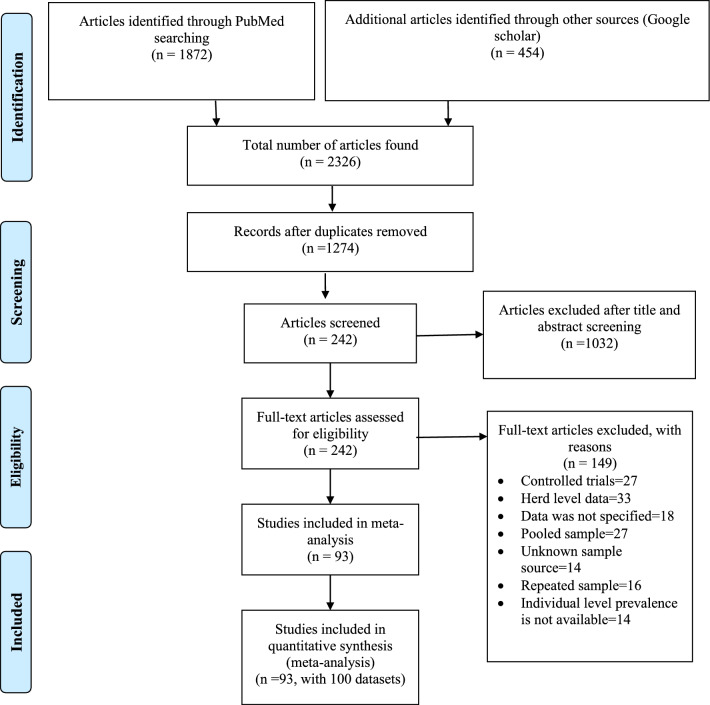
Study selection process for meta-analysis of *Salmonella*

Figure 1 shows the overall selection processes of the eligible articles used in this study. A total of 1872 articles were identified in the PubMed database and 454 articles in Google Scholar and CAB abstracts. We selected a total of 1274 articles after removal of duplicates or triplicates. After screening the titles and abstracts, only 242 papers were included. Finally, 93 articles (containing 100 datasets) were included to determine the prevalence of *Salmonella* in the South Asian Region.

### Overall Prevalence of *Salmonella*

**Fig. 2:**
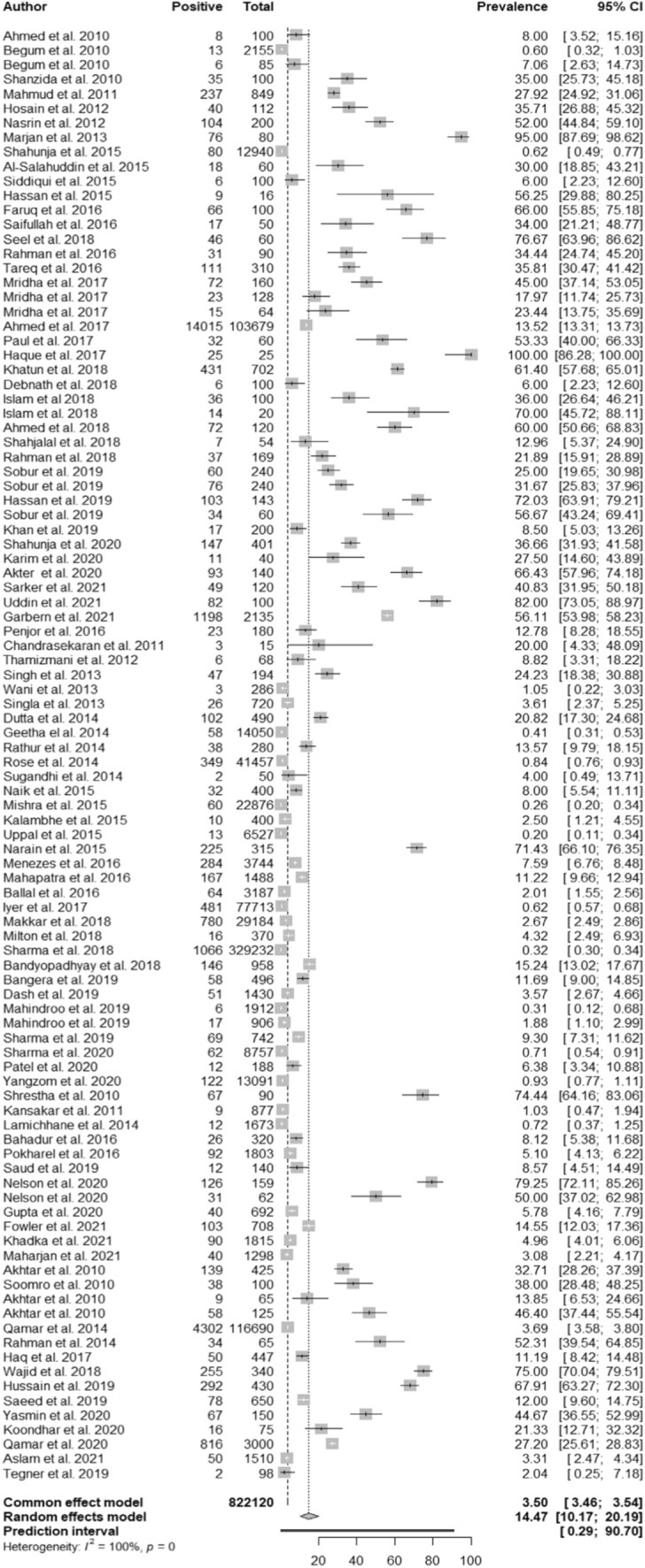
Forest plot of meta-analysis showing pooled prevalence of *Salmonella* in studies conducted in South Asia

The prevalence distributions of *Salmonella* in different categories are depicted in Table 1. A total of 100 datasets of 93 articles from South-Asian countries are presented for the prevalence of *Salmonella*. The overall prevalence of *Salmonella* in the random model effect was 14.47% (95% CI: 10.17–20.19) with a high degree of heterogeneity (*I*^2^ = 99.8%) (Fig. 2).

**Table 1:** Prevalence distribution of *Salmonella* in different categories

Variables (*P*-value)	Observations	Number of positive isolates	Sample size	No. of study	Sub-group analysis		
					Prevalence (%)	*Q*	*I*^2^
Country	Bangladesh (Ref)	17,558	126,607	41	34.23 (23.73–46.56)	6062.77	99.3%
	India	4375	561,526	31	3.28 (1.84–5.77)	9420.78	99.7%
	Pakistan	6204	124,072	14	26.15 (15.04–41.47)	6230.43	99.8%
	Nepal	648	9637	12	10.36 (3.73–25.63)	906.70	98.8%
	Bhutan	23	180	1	12.78 (8.64–18.50)	–	–
	Sri Lanka	2	98	1	2.04 (0.51–7.79)	–	–
Source	Animal (Ref)	2283	12,377	43	22.66 (15.41–32.03)	1926.99	97.8%
	Environment	1539	12,507	19	27.81 (12.01–52.07)	987.58	98.2%
	Human	24,988	797,236	38	5.81 (3.17–10.41)	35,583.25	99.9%
Locality	Both (Ref)	1200	4297	7	35.77 (20.91–54.00)	178.67	96.6%
	Rural	3415	399,544	35	8.85 (5.10–14.91)	9761.82	99.7%
	Urban	24,195	418,279	58	17.02 (10.42–26.56)	25,003.42	99.8%
Time	2010–2013 (Ref)	924	6646	19	17.88 (8.25–34.50)	779.49	97.7%
	2014–2017	20,990	411,997	36	11.02 (5.66–20.36)	16,282.64	99.8%
	2018–2021	6896	403,477	45	16.41 (10.08–25.58)	24,789.37	99.8%
Test	Culture (Ref)	5793	412,051	46	18.39 (10.44– 30.36)	24,540.96	99.8%
	PCR	1808	60,285	19	11.65 (5.62–22.60)	2394.48	99.2%
	Culture and Biochemical	20,394	247,994	25	12.00 (6.57–20.93)	9645.64	99.8%
	Culture and PCR	73	190	2	30.14 (2.80–86.62)	61.93	98.4%
	Biochemical	708	101,535	7	6.33 (1.44; 23.73)	1717.80	99.7%
	Antisera	34	65	1	52.31 (40.27; 64.09)	0.00	–

*Statistically significant (*P* value < 0.05)

#### Country

The highest prevalence of *Salmonella* in South Asia was observed in Bangladesh as 34.23% (95% CI: 23.73–46.56) with a high degree of heterogeneity (*I*^2^ = 99.3%). Pakistan also had a relatively higher prevalence of 26.15% (95% CI: 15.04–41.47). Only a single study was identified in Sri Lanka with the lowest prevalence (2.04%, 95% CI: 0.51–7.79) among South Asian countries. India also had a lower prevalence of 3.04% but a very high variation in between-study (*I*^2^ = 99.7%). Nepal (10.36%) and Bhutan (12.78%) both had a prevalence around the pooled prevalence of South Asia. Noteworthy, we did not find any articles on Maldives and Afghanistan in our search.

#### Source

Based on sample origin, we categorized the prevalence of *Salmonella* into Human, Animal, and Environment. The highest prevalence was found in environment (27.81%, 95% CI: 12.01–52.07, *I*^2^ = 98.2%) followed by animal-source (22.66%, 95% CI: 15.41–32.03, *I*^2^ = 97.8%) and human origin (5.81%, 95% CI: 3.17–10.41, *I*^2^ = 99.9%).

#### Locality

In this study, we considered *Salmonella* prevalence into three categories based on locality: rural, urban, and semi-urban areas. From rural areas, 35 studies demonstrated a high degree of heterogeneity (99.7%), and the prevalence was 8.85% (95% CI: 5.10–14.91). In the urban region, 58 studies out of 100 showed *Salmonella* positivity which was almost double (17.02%, 95% CI: 10.42–26.56) than rural areas. The prevalence of *Salmonella* was 35.77% (95% CI: 20.91–54.00) in semi-urban areas.

#### Time

The temporal pattern of *Salmonella* prevalence was irregular. It was 17.88% in 2010–2013, then decreased to 11.02% (2014–2017), and further increased to 16.41% in recent years (2018–2021).

#### Diagnostic Test

In this study, the variation of detection of *Salmonella* showed that some methods had higher rates than others. Using an antisera method resulted in the highest rate as 52.31% (95% CI: 40.27–64.09) of cases being detected in this way followed by combining two methods, culture and PCR as 30.14% (95% CI: 2.80–86.62). On the other hand, biochemical test had the lowest rate, 6.33% (95% CI: 1.44–23.73) of cases.

#### Source of Heterogeneity

To measure the prevalence of *Salmonella* in South Asia, two sources of heterogeneity were identified as significant in univariate meta-regression: Country (*P* < 0.0001) and Source (*P* < 0.0001). The heterogeneity of locality was borderline significant (*P* = 0.0547). In multivariate meta-regression (Table 2), both country and source were found to be significant. There was no significant correlation found between groups, thus no interactions were added in the multivariate model. In multivariate, the prevalence in India and Nepal were significantly different from others. Studies in which the sources of samples were humans were borderline significant (*P*-value = 0.0529) from other sources of samples.

**Table 2:** Final multivariate meta-analysis with different variables of *Salmonella*

Variables (*P*-value)	Observations	Univariate meta-regression		Multivariate meta-regression	
		Co-efficient	*P*-value	Co-efficient	*P*-value
Country (< 0.0001*)	Bangladesh (Ref)	–	–	–	–
	India	−2.73 (−3.50, −1.96)	< 0.0001	−0.22 (−0.35, −0.09)	0.0012*
	Pakistan	−0.38 (−1.38, 0.61)	0.4495	0.11 (−0.10, 0.31)	0.2993
	Nepal	−1.50 (−2.56, −0.44)	0.0054	0.004 (−0.19, 0.18)	0.9712
	Bhutan	−1.29 (−4.55, 1.97)	0.0041	−0.35 (−0.80, 0.09)	0.1212
	Sri Lanka	−3.43 (−7.00, 0.14)	0.0600	−0.36 (−0.81, 0.08)	0.1076
Source (0.0001*)	Animal (Ref)	–	–	–	–
	Environment	0.26 (−0.77, 1.28)	0.6253	−0.004 (−0.13, 0.12)	0.9500
	Human	−1.55 (−2.37, −0.73)	0.0002	−0.09 (−0.21, 0.02)	0.1038
Locality (0.0547*)	Both (Ref)			–	–
	Rural	−1.76 (−3.37, −0.15)	0.0325	−0.09 (−0.29, 0.12)	0.4065
	Urban	−1.01 (−2.57, 0.54)	0.2021	0.008 (−0.18, 0.19)	0.9315
Time (0.4953)	2010–2013 (Ref)	–	–	–	–
	2014–2017	−0.57 (−1.70, 0.56)	0.3252		
	2018–2021	−0.10 (−1.20, 0.99)	0.8535		
Test (< 0.0001*)	PCR (Ref)	–	–	–	–
	Culture	0.19 (−0.04, 0.24)	0.1435	0.07 (−0.05, 0.20)	0.2781
	Culture and biochemical	0.002 (−0.15, 0.15)	0.9768	−19 (−0.39, 0.010)	0.0615
	Culture and PCR	0.19 (−0.18, 0.57)	0.3173	−0.01 (−0.35, 0.33)	0.9575
	Biochemical	−0.04 (−0.27, 0.18)	0.7056	−0.05 (−0.24, 0.14)	0.6247
	Antisera	0.32 (−0.22, 0.85)	0.2420	0.11 (−0.40, 0.61)	0.6877

*Statistically significant (*P* value < 0.05)

### Pooled Prevalence and Distribution of *Salmonella* Serotypes

Table 3 shows the pooled prevalence and distribution of *Salmonella* serotypes. The prevalence of *S. enterica* was 14.22% (95% CI: 4.02–39.64) followed by *S.* *pullorum* 13.50% (95% CI: 5.64–29.93), *S. indica* 5.05% (95% CI: 0.52–35.19), *S. salamae* 3.74% (95% CI: 2.08–6.63), *S.* *typhi* 3.24% (95% CI: 1.58–6.51), *S.* Paratyphi B 2.66% (95% CI: 1.72–4.10), *S.* *typhimurium* 2.43% (95% CI: 0.73–7.74), and *S. houtenae* 2.06% (95% CI: 0.78–5.36) (Table 3). There is a 20.88% (95% CI: 13.17–31.49) prevalence of non-specific *Salmonella* in the population. The prevalence of the remaining *Salmonella* species is less than 2%. Prevalence of different *Salmonella* serovar in different countries are depicted in supplementary Fig. 1. The prevalence of *Salmonella* varies considerably between different sources (Table 3). In human cases, non-specific *Salmonella* were observed at 3.06% followed *by S. enterica* (2.92%). *S. enterica* was highly prevalent in animals (34.71%) followed by *S.* *pullorum* (22.67%). Considering environmental sources, non-specific *Salmonella* was observed as 33.13% followed by *S.* *enteritidis* (16.00%).

**Table 3:** Pooled prevalence and distribution of *Salmonella* serotypes

Species	No. of study	Prevalence % (95% CI)	*Q*	*I*^2^	Source-specific prevalence % (95% CI)		
					Human	Animal	Environment
*S.* t*yphi*	27	3.24 (1.58–6.51)	13,329.44	99.8%	1.48 (1.45–1.51)	4.08 (2.54–6.41)	9.25 (8.28–10.32)
*S.* Paratyphi A	18	1.18 (0.56–2.48)	4884.89	99.7%	0.59 (0.57–0.61)	1.00 (0.05–6.24)	3.07 (2.49–3.76)
*S.* Paratyphi B	4	2.66 (1.72–4.10)	13.25	77.4%	1.81 (1.18–2.74)	3.33 (1.36–7.45)	4.07 (3.40–4.85)
*S.* *typhimurium*	7	2.43 (0.73–7.74)	193.72	96.9%	0.09 (0.04–0.21)	3.66 (2.82–4.73)	10.55 (9.50–11.70)
*S. enterica*	7	14.22 (4.02–39.64)	615.17	99.0%	2.92 (2.40–3.55)	34.71 (31.01–38.58)	14.42 (9.69–24.98)
*S.* *enteritidis*	5	1.24 (0.10–13.57)	78.09	94.9%	0.011 (0.001–0.07)	4.27 (2.97–6.08)	16.00 (9.70–24.99)
*S.* *pullorum*	2	13.50 (5.64–29.93)	9.64	89.6%	–	22.67 (16.41–30.36)	7.00 (3.10–14.38)
*S.* Kentucky	4	1.17 (0.34–4.00)	192.35	98.4%	0.42 (0.31–0.57)	4.19 (3.29–5.30)	–
*S.* Virchow	3	0.73 (0.21–2.46)	34.82	94.3%	0.21 (0.13–0.33)	1.67 (0.95–2.85)	–
*S. indica*	2	5.05 (0.52–35.19)	9.30	89.2%	–	5.05 (0.52–35.19)	–
*S. salamae*	2	3.74 (2.08–6.63)	0.03	0.0%	–	3.74 (2.08–6.63)	–
*S.* Weltevereden	1	0.011 (0.001–0.08)	0.0	–	0.011 (0.001–0.08)		
*S.* Bareilly	1	0.011 (0.001–0.08)	0.0	–	0.011 (0.001–0.08)	–	–
*S.* Stanley	1	0.011 (0.001–0.08)	0.0	–	0.011 (0.001–0.08)	–	–
*S.* Worthington	1	0.011 (0.001–0.08)	0.0	–	0.011 (0.001–0.08)	–	–
*S. houtenae*	1	2.06 (0.78–5.36)	0.0	–	–	2.06 (0.78–5.36)	–
*S. bongori*	1	1.00 (0.14–6.75)	0.0	–	–	1.00 (0.14–6.75)	–
*S.* Choleraesuis	1	1.00 (0.14–6.75)	0.0	–	–	1.00 (0.14–6.75)	–
Nonspecific *Salmonella*	56	20.88 (13.17–31.49)	13,425.59	99.6%	3.06 (2.94–3.19)	14.46 (13.67–15.28)	33.13 (31.35–34.96)

### The Prevalence of Antibiotic Resistance in *Salmonella* Species

This study investigated the antimicrobial resistance rates of different *Salmonella* serotypes (Table 4). *S.* *pullorum* had a high resistance rate of 90.06% (95% CI: 5.96–99.92) followed by *S. enterica* as 86.26% (95% CI: 50.07–97.51). However, due to the paucity of studies on these serotypes, limited data were available for less common strains such as *S.* Weltevereden, *S.* Bareilly, *S.* Stanley, *S.* Worthington, and *S. houtenae*, with a resistance rate of 0.00%. *S.* Virchow had the highest level of antimicrobial resistance among human cases with a significant higher rate of 80% (95% CI: 44.22–96.46). *S.* *pullorum* had the highest resistance rate in animals, reaching 100% (95% CI: 69.87–100). Notably, *S. enterica* exhibited a startlingly high resistance rate of 100% (95% CI: 71.66–100) in environmental samples. We analyzed the resistance status of *Salmonella* against different antibiotics (Supplementary Table 1) of which most tested antibiotics are presented (Table 5). Table 5 shows the highest pooled percentage of *Salmonella*-resistance against nalidixic acid (74.25%) followed by tetracycline (37.64%), trimethoprim/sulfamethoxazole (32.92%), amoxicillin (32.18%), azithromycin (31.05%), chloramphenicol (22.45%), gentamicin (15.94%), and ampicillin (12.12%). Conversely, the lowest percentage of resistance was found against ceftriaxone (1.07%) followed by cefixime (1.24%), co-trimoxazole (3.92%), and ciprofloxacin (7.58%).

**Table 4:** Prevalence of AMR percentage in *Salmonella* species depending on source-specific samples

Species	No. of study	AMR Prevalence (95% CI)	*Q*	*I*^2^	Source-specific AMR prevalence (95% CI)		
					Human	Animal	Environment
*S.* *typhi*	27	67.70% (57.51–76.45)	38.50	32.5%	67.96% (61.06–74.18)	40% 19.98–63.59)	60.53% (43.45–75.51)
*S.* Paratyphi A	18	54.59% (39.74–68.64)	30.57	44.4%	49.63% (40.96–58.32)	50% (23.66–76.34)	76.34% (69.23–38.88)
*S.* Paratyphi B	4	59.94% (40.11–76.97)	4.74	36.7%	60.71% (40.73–77.87)	66.67% (24.11–94.0)	61.54% (32.28–84.87)
*S.* *typhimurium*	7	70.57% (58.03–80.61)	7.93	24.3%	66.67% (24.11–94)	75% (61.88–84.89)	59.26% (39.01–76.99)
*S. enterica*	7	86.26% (50.07–97.51)	8.90	32.6%	39.13% (20.47–61.22)	90.48% (68.17–98.33)	100% (71.66–100)
*S.* *enteritidis*	5	60.37% 26.46–86.57)	8.01	50.1%	40% (13.69–72.63)	64.29% (47.99–78.00)	42.86% (18.81–70.35)
*S.* *pullorum*	2	90.06% (5.96–99.92)	0.00	0.00%	–	100% (69.87–100)	42.86% (18.81–70.35)
*S.* Kentucky	4	53.42% (7.84–93.93)	4.83	37.9%	41.18% (19.43–66.55)	68.42% (43.50–86.44)	–
*S.* Virchow	3	66.11% (0.12–99.97)	0.00	0.0%	80% (44.22–96.46)	52.17% (31.08–72.58)	–
*S. indica*	2	44.44% (23.99–66.96)	0.28	0.0%	–	44.44% (23.99–66.96)	–
*S. salamae*	2	44.59% (6.02–90.99)	6.28	84.1%	–	50% (29.03–70.97)	–
*S.* Weltevereden	1	0.00	0.00	–	0.00	–	–
*S.* Bareilly	1	0.00	0.00	–	0.00	–	–
*S.* Stanley	1	0.00	0.00	–	0.00	–	–
*S.* Worthington	1	0.00	0.00	–	0.00	–	–
*S. houtenae*	1	25.00% (6.30–62.29)	0.00	–	–	25.00% (6.30–62.29)	–
*S. bongori*	1	50.00% (22.45–77.54)	0.00	–	–	50.00% (22.45–77.54)	–
*S.* Choleraesuis	1	10.00% (1.39–46.72)	0.00	–	–	10.00% (1.39–46.72)	–
Nonspecific *Salmonella*	52	83.75% (75.02–89.83)	47.19	0.0%	74.44% (63.97–82.80)	73.90% (68.42–78.74)	83.110% (75.69–88.66)

**Table 5:** Resistance percentage of *Salmonella* against most used antibiotics

Antibiotics	Number of samples tested	Number of resistant	Percentage of resistance with 95% CI
Ampicillin	40,216	4875	12.12 (11.80–12.45)
Amoxycillin	3297	1061	32.18 (30.59–33.81)
Azithromycin	4840	1503	31.05 (29.75–32.38)
Ciprofloxacin	37,196	2820	7.58 (7.31–7.86)
Ceftriaxone	38,386	410	1.07 (0.97–1.18)
Cefixime	36,046	446	1.24 (1.13–1.36)
Chloramphenicol	13,580	3049	22.45 (21.75–23.16)
Cotrimoxazole	33,735	1323	3.92 (3.72–4.13)
Gentamicin	2911	464	15.94 (14.63–17.32)
Tetracycline	2858	1076	37.65 (35.87–39.45)
Nalidixic acid	4819	3578	74.25 (72.99–75.48)
Trimethoprim/sulfamethoxazole	6392	2104	32.92 (31.76–34.08)

Among different classes of antibiotics, the highest resistance percentage was against fluoroquinolone (67.67%). On the other hand, the lowest resistance percentage was in aminoglycoside (0.25%) followed by macrolide (0.83%) (Supplementary Table 1). The resistance pattern of specific *Salmonella* serotypes according to different antibiotics is described in Table 6. The resistance rate of *S.* *typhi* to nalidixic acid was 88.12% (95% CI: 62.58–97.05), highlighting the antibiotic's limited efficacy against this serotype. Similarly, *S.* Paratyphi A was resistant to nalidixic acid at a rate of 91.32% (95% CI: 78.25–96.85). Resistance to cefixime reached 60% (95% CI: 20.04–89.97) in *S.* Paratyphi B which indicate moderate resistance. Furthermore, *S.* *typhimurium* was resistant to tetracycline at 87.80% (95% CI: 73.85–94.83).

**Table 6:** AMR pattern of *Salmonella* serotypes against different antibiotics

Serovar	AMR percentage (95% confidence interval)											
	Ampicillin	Amoxycillin	Azithromycin	Ciprofloxacin	Ceftriaxone	Cefixime	Chloramphenicol	Cotrimoxazole	Gentamicin	Tetracycline	Nalidixic acid	Trimethoprim/sulfamethoxazole
*S.* *typhi*	33.28 (15.22–58.06)	9.10 (1.46–40.32)	2.76 (0.29–21.58)	12.16 (3.73–33.09)	0.08 (0.006–0.99)	0.11 (0.005–2.27)	12.81 (4.89–29.58)	13.42 (6.86–24.59)	2.77 (0.35–18.70)	50.72 (19.37–81.51)	88.12 (62.58–97.05)	83.99 (21.92–98.99)
*S.* Paratyphi A	2.95 (0.76–10.78)	17.20 (1.60–72.60)	6.64 (4.22–10.29)	12.65 (2.22–48.00)	0.009 (0.0001–1.53)	0.008 (0.00–2.15)	2.24 (0.66–7.28)	2.55 (0.85–7.39)	6.47 (1.92–19.63)	8.81 (0.00–99.98)	91.32 (78.25–96.85)	32.39 (2.84–88.70)
*S.* Paratyphi B	10.61 (7.33–15.13)	–	40 (10.02–79.96)	3.26 (1.64–6.39)	6.21 (0.09–82.08)	60 (20.04–89.97	7.19 (2.20–21.10)	4.31 (0.28–41.59)	2.41 (0.04–59,82)	–	19.42 (12.88–28,19)	
*S.* *typhimurium*	67.24 (41.25–85.71)	–	9.37 (3.05–25.35)	57.23 (18.85–88.52)	6.25 (1.57–21.81)		9.06 (6.52–12.48)	–	28.60 (5.61–72.99)	87.80 (73.85–94.83)	66.41 (43.60–83.49)	5.20 (0.91–24.67)
*S. enterica*	16.72 (0.001–99.97)	0.00	2.71 (0.75–9.25)	9.66 (6.98–13.21)	0.70 (0.05–8.81)	0.00	4.52 (0.97–18.57)	–	1.67 (0.03–45.31)	86.59 (34.33–98.76)	85.15 (35.54–98.35)	
*S.* *enteritidis*	93.74 (62.03–99.27)	–	20 (2.72–69.10)	34.72 (1.25–95.73)	30.77 (16.19–50.55)	14.28 (4.68–36.13)	2.78 (0.01–85.77)	–	27.44 (1.36–91.19)	79.76 (53.69–93.05)	85.02 (36.78–98.23)	6.25 (0.87–33.54)
*S.* *pullorum*	100	–	–	16.46 (0.27–93.35)	23.53 (12.23–40.46)	23.53 (12.22–40.46)	16.59 (0.19–95.39)	–	16.50 (0.22–94.45)	82.64 (17.94–99.04)	91.50 (62.76–98.57)	28.57 (7.20–67.33)
*S.* Kentucky	86.12 (62.35–95.87)	–	0.00	97.36 (92.16–99.15)	0.00		0.00	0.00	25.64 (14.39–41.64)	100	97.78 (61.51–99.92)	–
*S.* Virchow	0.00	0.00	–	44.47 (21.32–70.13)	5.56 (0.78–30.65)		5.55 (0.77–30.65)	0.00	83.33 (59.14–94.52)	100	100	0.00
*S. indica*	11.11 (4.23–26.11)	–	–	0.00	–		0.00	2.77 (0.38–17.25)	0.00	0.00	–	–
*S. salamae*	100	–	–	0.00	–		0.00	0.00	0.00	0.00		–
*S.* Weltevereden	0.00	–	–	0.00	0.00		0.00	0.00	0.00	–	0.00	–
*S.* Bareilly	0.00	–	–	0.00	0.00		0.00	0.00	0.00	–	0.00	–
*S.* Stanley	0.00	–	–	0.00	0.00		0.00	0.00	0.00	–	0.00	–
*S.* Worthington	0.00	–	–	0.00	0.00		0.00	0.00	0.00	–	0.00	–
*S. houtenae*	25 (3.35–76.21)	–	–	0.00	–		0.00	0.00	0.00	25 (3.35–76.21)	–	–
*S. bongori*	–	–	–	–	–		–	–	–	–	–	–
*S.* Choleraesuis	–	–	–	–	–		–	–	–	–	–	–
Nonspecific *Salmonella*	61.19 (35.02–82.18)	44.06 (22.83–67.70)	60.77 (23.16–88.84)	12.58 (5.88–24.86)	8.59 (2.53–25.39)	46.75 (25.04–69.77)	7.08 (1.92–22.90)	13.29 (6.45–25.42)	8.96 (4.46–17.15)	49.42 (18.23–81.06)	38.45 (10.77–76.37)	32.51 (13.85–59.08)

*–* No study found

### Overall Antimicrobial Drug Resistance Percentage

In this present paper, the overall antimicrobial drug resistance percentage was 70% (95% CI: 63.0–76.0) with a heterogeneity of 23.6%. The overall antimicrobial drug percentage of *Salmonella* with different variables are depicted in Table 7. The overall antimicrobial resistance patterns of different antibiotics are also given in Supplementary Table 1.

**Table 7:** Overall antimicrobial drug resistance in *Salmonella* in different categories

Variables (*P*-value)	Observations	No. of study	Sub-group analysis			Univariate Meta-regression	
			Prevalence	*Q*	*I*^2^	Coeff	*P*-value
Source (0.0316*)	Animal (Ref)	56	77% (65–86)	75.24	26.9%	–	
	Environment	23	78% (65–88)	21.2	0.0%	0.24 (−0.63, 1.12)	0.5884
	Human	61	60% (50–69)	84.23	28.8%	−0.72 (−1.37, 0.06)	0.0315
Locality (0.005*)	Semi-urban (Ref)	13	96% (79–99)	1.69	0.0%	–	
	Rural	43	61% (47–73)	64.80	35.2%	−2.40 (−3.61, −1.18)	0.0001
	Urban	84	69% (60–76)	105.01	21.0%	−2.03 (−3.19, −0.88)	0.0006
Time (0.0756)	2010–2013 (Ref)	23	53% (41–65)	40.83	46.1%	–	
	2014–2017	44	68% (58–76)	65.66	34.5%	0.69 (−0.19, 1.57)	0.1259
	2018–2021	73	77% (66–86)	68.52	0.0%	0.94 (0.13, 1.76)	0.023

*Statistically significant (*P* value < 0.05)

#### Source

The prevalence of overall antimicrobial resistance percentage was highest in the samples from the environmental origin (78%, 95% CI: 65.0–88.0), with a zero heterogeneity (*I*^2^ = 0.00%), followed by samples from animal sources (77%, 95% CI: 65.0–86.0, *I*^2^ = 26.9%) and human origin (60%, 95% CI; 50.0–69.0, *I*^2^ = 28.8%) (Table 7).

#### Locality

The drug resistance of semi-urban areas was 96% (95% CI: 79.0–99.0, *I*^2^ = 0.0%), meaning that 96% of all *Salmonella* are resistant to antimicrobials. Rural and urban areas had a resistance of 61% (95% CI: 47.0–73.0, *I*^2^ = 35.2%) and 69% (95% CI: 60.0–76.0, *I*^2^ = 21%), respectively.

#### Time

The temporal distribution of the overall antimicrobial resistance percentage against *Salmonella* shows an increasing pattern. It was 53% in between 2010 and 2013, then increased to 68% in 2014–2017, and finally rose to 77% in 2018–2021.

#### Source of Heterogeneity in Overall Antimicrobial Resistance Percentage

In univariate meta-regression, two sources of heterogeneity were identified as significant in the occurrence of the overall antimicrobial resistance: Source (*P* = 0.0315) and Locality (*P* = 0.005). The group time was found borderline significant (*P* = 0.0756); thus, it was included in the multivariate meta-regression. No interactions were included in the multivariate due to a lack of significant correlation among them. In multivariate meta-regression (Table 8), source, locality, and time were found to be significant. Samples of human origin (*P* = 0.0083) were significantly different from animal and environmental sources. Considering locality, the overall antimicrobial resistance in both rural (*P* = 0.0022) and urban areas (*P* = 0.0112) varied significantly from the semi-urban area. In the case of time, drug resistance varied significantly in recent years than the past.

**Table 8:** Final multivariate meta-regression analysis with different variables of antimicrobial-resistant *Salmonella*

Variables	Observations	Co-efficient	P-value
Source	Animal (Ref)	–	
	Environment	0.02 (−0.12, 0.16)	0.7750
	Human	−0.13 (−0.24, −0.02)	0.0188*
Location	Semi-urban (Ref)	–	
	Rural	−0.18 (−0.38, −0.006)	0.0576
	Urban	−0.12 (−0.29, 0.04)	0.1466
Time	2010–2013 (Ref)	–	
	2014–2017	0.19 (0.04, 0.33)	0.0155*
	2018–2021	0.17 (0.03, 0.30)	0.0148*
Test	PCR (Ref)	–	
	Culture	0.07 (−0.05, 0.20)	0.2565
	Culture and biochemical	0.09 (−0.05, 0.23)	0.2257
	Culture and PCR	0.15 (−0.08, 0.38)	0.1909
	Biochemical	0.07 (−0.12, 0.26)	0.4636
	Antisera	0.29 (−0.28, 0.86)	0.3191

*Statistically significant (*P* value < 0.05)

### Publication Bias

**Fig. 3:**
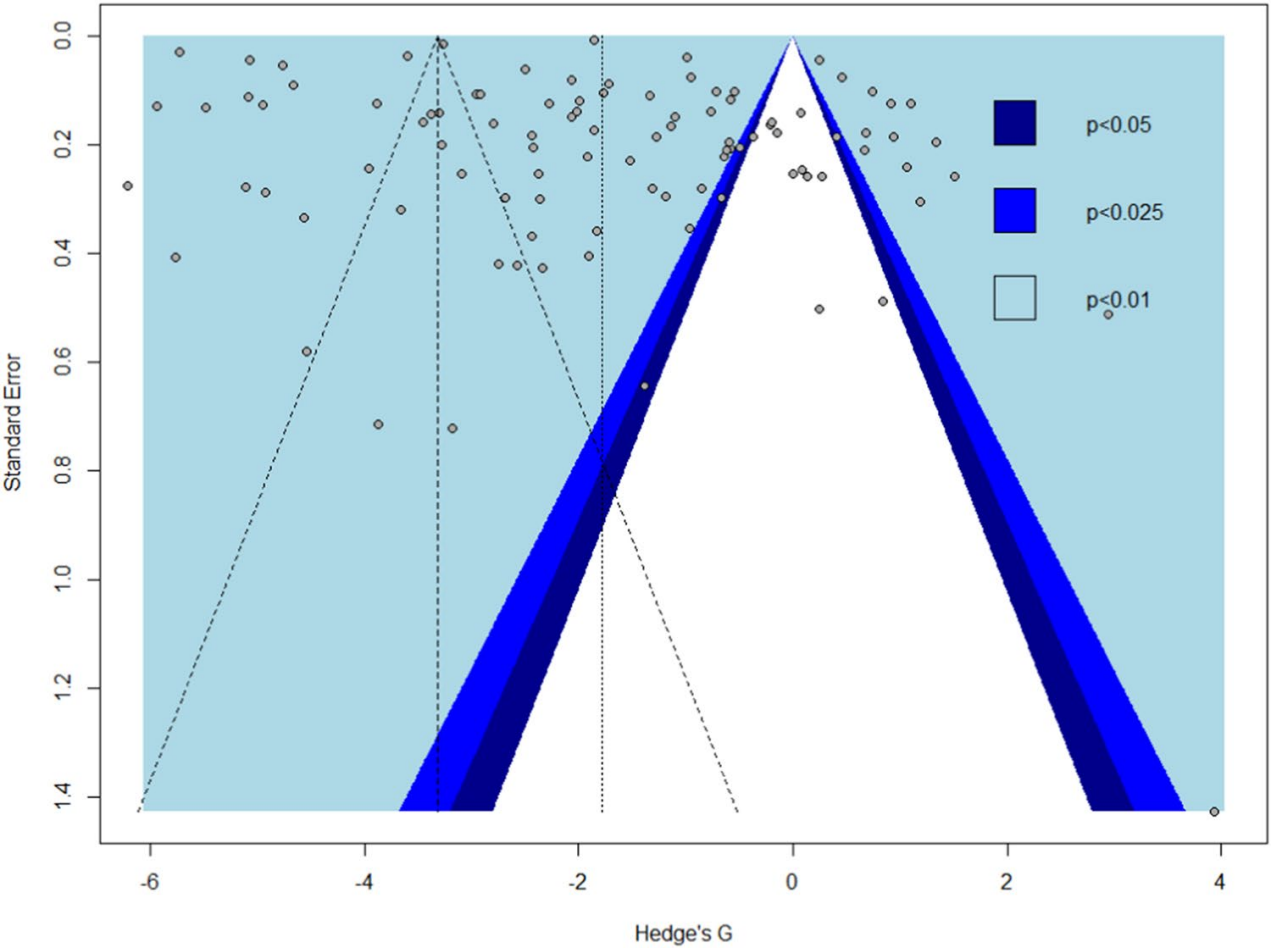
Funnel plot of meta-analysis showing publication bias in studies reporting the prevalence of *Salmonella*

The funnel plot indicates that there are some publication biases present in this meta-analysis (Fig. 3), however, Egger’s test result indicates that the funnel plot asymmetry is not significant (*P* = 0.3130). Thus, we can reject the concern of significant publication bias which might mask the original prevalence of *Salmonella*. The contour funnel indicates a substantial contribution of the studies to the overall meta-analysis in different levels of significance.

## Discussion

AMR is a worldwide health concern affecting significantly low- and middle-income countries, though high-income countries are also facing the threat of AMR at an alarming level. Antibiotic-resistant bacterial infections caused an estimated 1.2 million deaths in 2019, more deaths than AIDS or malaria [26]. *Salmonella* is globally one of the leading causes of human death among diarrheal diseases. Understanding the epidemiological status of *Salmonella* is thus crucial for controlling this pathogen [27]. This meta-analysis focuses on a comprehensive and robust assessment of current primary research on the prevalence of *Salmonella* in humans, animals, and the environment and its antimicrobial resistance status in South Asia.

This study indicates that the pooled prevalence of *Salmonella* was 14.47% (95% CI: 10.17–20.19) in South Asia. The subgroup analysis indicates the highest pooled prevalence of *Salmonella* in Bangladesh (34.24%, 95% CI: 23.73–46.56) followed by Pakistan (26.15%, 95% CI: 15.04–41.47), and Nepal (10.36%, 95% CI: 3.73–25.63). On the other hand, India had the lowest prevalence of *Salmonella* (*P*-value < 0.0001). This variation might be due to the difference in temperature, humidity, and other weather-related events [28], food habits [29], and availability of migratory birds [30]. The number of studies included might be a possible reason for the country-wise pooled prevalence variation. For instance, only a single study was found in Sri Lanka and Bhutan which may not reflect the actual pooled prevalence. The pooled prevalence of *Salmonella* among human samples was 5.81% (95% CI: 3.17–10.40). A study in the Middle East and Northern Africa reported almost similar pooled prevalence of *Salmonella* in humans [31]. The prevalence of *Salmonella* in humans was lower than in animals (22.66%, 95% CI: 15.41–32.03) and in the environment (27.81%, 95% CI: 12.01–52.07). One of the possible reasons for the comparatively higher prevalence of *Salmonella* in the environment is that they can survive both in soil and water and even may transfer with flies from different hosts to the environment [32]. Moreover, not all the *Salmonella* species can transmit from the environment to animals as well as animals to humans [33]. Additionally, unhygienic animal husbandry practices were recorded in South Asia which may be a potential cause of higher prevalence in animals [34, 35]. When categorizing into regions, the prevalence of *Salmonella* was found higher in semi-urban regions (35.77%, 95% CI: 8.25–34.50) than in the rural (8.85%, 95% CI: 5.10–14.91) and urban regions (17.02%, 95% CI: 10.42–26.56). Though rural communities have higher exposure to zoonotic pathogens due to frequent contact with wild and domesticated animals, there are limited disease surveillance programs in rural areas, and they receive less healthcare support [36]. Nevertheless, we found comparatively less prevalence in rural areas than in semi-urban and urban regions. This might be due to high population density in urban areas [37] which leads to greater transmission of infectious agents [38]. This meta-analysis found 18 different types of *Salmonella* serovar. This study found that enteric *Salmonella* is more evident than other serovars in South Asia. Some other serovars like *S.* Kentucky*, S. salamae, S. houtenae*, etc. are also present in fewer numbers, however, they may arise as a significant threat [39, 40]. The transmission of *Salmonella enterica* Serovar Kentucky has a travel association and Southeast Asia is at major risk [41]. Likewise, local *S*. *typhi* and *S*. Paratyphi A strains were identified in Nepal which had close genetic relatives in other South Asian countries, which highlights a major public health concern with inter and intra-country transmission [42].

*Salmonella* has a broad-spectrum host range, hence is considered as a universal pathogen. Each serovar has a different ability to adapt to the host environment and cause virulency. Some *Salmonella* serovars are restricted within one host whereas some have broad host spectrum [43]. For instance, *Salmonella enterica* serovars were isolated in majority of cases (99.5%) from animals and humans [43]. With no exception, in this study, we found that *Salmonella enterica* serovar was highly prevalent in humans (2.92%), animals (34.71%) and the environment (14.42%). Similarly, *S.* *enteritidis* and *S.* *typhimurium* were observed in all source-specific samples, even though not highly prevalent. On the other hand, *S.* *pullorum* is known to host-restrictive serovar only in poultry as primary host [44]. Of note, in this study, no human cases of *S.* *pullorum* are found but highly prevalent in animal-specific samples (22.67%). Therefore, the ability of a pathogen to spread disease in populations in many respects is influenced by host adaptation. Asymptomatic animals can shed the bacterium continually via feces, and *Salmonella* regardless of serovar can persist in dry environments as well as in water for many weeks to months. These animals can contaminate environment and directly transmit pathogens to susceptible hosts [44]. Thus, in general sense, environment-specific samples may serve as a good source of respective bacterium and become a risk to susceptible hosts.

We analyzed the resistance percentage of *Salmonella* against different antibiotics. In the present study, the resistance of *Salmonella* to nalidixic acid was 74.25%, fluoroquinolone 67.67%, tetracycline 37.64%, trimethoprim/sulfamethoxazole 32.92%, and amoxicillin 32.18%. There was an increasing trend in the prevalence (53–77%) of overall antimicrobial-resistant *Salmonella* from 2010–2013 to 2018–2021. Indiscriminate application of antibiotics in human and animal health, and food production and subsequently leaching of the antibiotics into the environment are contributing to the increased AMR bacteria [22]. In South Asia, *S*. *typhi* and *S*. Paratyphi were reported as endemic, and several antibiotics were used for enteric fever resulting in the development of antimicrobial resistance by these antibiotics [45]. Due to the resistance of most *Salmonella* species to first-line antibiotics in clinical cases, critically important antibiotics such as fluoroquinolones, third and fourth generation cephalosporins, macrolide, etc. have become a choice for the treatment of invasive *Salmonella* infections [8, 46]. As a consequence of increasing resistance, WHO enlisted fluoroquinolone-resistant *Salmonella* spp. in the list of priority pathogens describing the urgent need for antibiotics against these bacteria [8].

In our result, the variability in the prevalence of *Salmonella* is strongly evident (*I*^2^ > 96%). A meta-regression model was used to assess the influence of different variables as well as methodological variation on the prevalence estimation of *Salmonella*. Different variables i.e., different sources of sample, country, and location might be responsible for the between-study variation which is obvious in our final multivariable model. Different countries and locations have diverse geographic patterns, seasonal variations, different food habits of people, and economic disparities which are some contributing factors to the variance in the prevalence of *Salmonella* [47]. Furthermore, different sample sources, for instance, environmental samples are often affected by various climatic events (e.g., temperature and rainfall), water temperature, soil moisture, soil types, presence of plants, etc. Again, types of animal farms may also add variations in the prevalence. For example, *Salmonella* was reported to be identified more frequently in swine farms compared to dairy and poultry farms [48]. The difference in the estimation of the prevalence may be partly due to the sorts of specimens obtained, for instance, blood culture, rectal swabs, feces from intestines, feces from rectums, voided feces, and mixed samples. On the other hand, global heterogeneity in the prevalence of *Salmonella* in animals was detected which may be due to the variation in the methodological procedures used in the isolation and identification of the organism (Supplementary Data). For instance, the isolation of *Salmonella* from whole feces may be different from the isolation from fecal swabs in terms of sensitivity. Also, culture-based testing of bacteria may not often reflect the actual prevalence (i.e., bacteria may present as a viable but non-culturable state) which might also add variation in the pooled prevalence [48]. With that in mind, random-effect model analysis is usually recommended in this situation assuming real differences in sampling variability.

The funnel plot was used to quantify and illustrate the extent of publication bias in the selected studies. The funnel is evidently not symmetrical and some of the points fall outside of it which indicates the presence of publication bias. The sources of the funnel plot asymmetry were tested by the Egger test to confirm the small study effects. The estimated bias coefficient was 6.79 with a standard error of 0.084 (*P* = 0.31). As a result, the test reveals a deficiency of evidence supporting the small study effects present. However, there are many different possible factors for funnel plot asymmetry, namely selection bias, true heterogeneity, data irregularities, artifacts as well as by-chance [49]. In this meta-analysis, unpublished papers, conference abstracts, and government reports were excluded since they seldom contain enough information to allow for relevant screening, data extraction, and analysis.

## Conclusion

In conclusion, the pooled prevalence of *Salmonella* was 14.47% from 2010 to 2021 in South Asia. The random effects pooled prevalence in Bangladesh, India, Pakistan, Nepal, Bhutan, and Sri Lanka were 34.24%, 3.28%, 26.15%, 10.36%, 12.78%, and 2.04% respectively, although there was heterogeneity between studies in most of these regions. The prevalence of overall antimicrobial resistance *Salmonella* was increased from 2010–2013 to 2018–2021 as 53–77%. Resistance to quinolones, tetracycline, trimethoprim/sulfamethoxazole, and amoxicillin was comparatively higher. This study indicates that the prevalence of AMR Salmonella is increasing with time in South Asia, and there are multiple potential reasons for this. Therefore, proper use of antimicrobials, regular surveillance of AMR, implementing antibiotic stewardship, and policy making, and implementation is necessary for controlling the spread of antibiotic resistant bacteria.

### Supplementary Information

Below is the link to the electronic supplementary material.

Supplementary file1 (DOCX 37 KB)

Supplementary file2 (XLSX 46 KB)

Supplementary file3 (XLSX 60 KB)

Supplementary file4 (DOCX 374 KB)

## Data Availability

All data generated and analyzed during this study are included in the main manuscript or supplementary files.

## Abbreviations

AIDS: Acquired immunodeficiency syndrome (AIDS)
AST: Antimicrobial susceptibility testing
AMR: Antimicrobial resistant
CDC: Centers for disease control and prevention
CI: Confidence interval
GDP: Gross domestic product
HIV: Human immunodeficiency virus
NTS: Non-typhoidal Salmonella (NTS)
PCR: Polymerase chain reaction
PRISMA: Preferred reporting items for systematic reviews and meta-analyses
USA: United States of America
WHO: World Health Organization
