# Supplementary material for: Prevalence and Antimicrobial Resistance Profile of Salmonella Isolated from Human, Animal and Environment Samples in South Asia: A 10-Year Meta-analysis
Source: J Epidemiol Glob Health. 2023 Oct 26;13(4):637–52. doi: 10.1007/s44197-023-00160-x (PMC10686918; doi:10.1007/s44197-023-00160-x)
Supplement: Supplementary file 1 — Supplementary file1 (DOCX 37 KB) [file 44197_2023_160_MOESM1_ESM.docx]

**Prevalence and antimicrobial resistance profile of *Salmonella* isolated from human, animal and environment samples in South Asia: a 10-year meta-analysis**

**Himel Talukder^1,2^, Shamsul Alam Roky**^3^**, Konad Debnath^1,4^, Binayok Sharma**^5,6^**, Juned Ahmed ^7,8^ and Sawrab Roy^9,10^**

*^1^Department of Epidemiology and Public Health, Sylhet Agricultural University, Bangladesh. ^2^Department of Geography and Environmental Sustainability, University of Oklahoma, USA. ^3^Department of Dairy Science, Faculty of Veterinary, Animal, and Biomedical Sciences, Sylhet Agricultural University, Sylhet, 3100, Bangladesh. ^4^International Centre for Diarrheal Disease Research, Bangladesh. ^5^Department of Medicine, Sylhet Agricultural University, Bangladesh. ^6^Department of Animal Science, Purdue University, USA. ^7^Department of Pathology, Sylhet Agricultural University, Bangladesh. ^8^School of Veterinary Medicine, Texas Tech University, Amarillo, TX-79106, USA. ^9^Department of Microbiology and Immunology, Sylhet Agricultural University, Bangladesh. ^10^Department of Veterinary Pathobiology, College of Veterinary Medicine, University of Missouri Columbia, MO, USA.*

^3,4,5,6^ authors (*Shamsul Alam Roky, Konad Debnath, Binayok Sharma*) contributed equally.

**Corresponding author:**

Sawrab Roy (sawrab.sau@gmail.com)

**Supplementary Table 1** Resistance percentage of Salmonella against antibiotic

| **Antibiotics** | **Total Number of sample tested** | **Total Number of resistant** | **Percentage of resistance** |
| --- | --- | --- | --- |
| Ampicillin | 40216 | 4875 | 12.12 |
| Amoxycillin | 3297 | 1061 | 32.18 |
| Amoxyclave | 111 | 14 | 12.61 |
| Amikacin | 753 | 98 | 13.01 |
| Amoxycillin and clauvonic acid | 551 | 211 | 38.29 |
| Amoxycillin and salbactam | 38 | 3 | 7.89 |
| Aztreonam | 195 | 5 | 2.56 |
| Azythromycin | 4840 | 1503 | 31.05 |
| Carbenicillin | 6 | 6 | 100 |
| Cephalothin | 1543 | 91 | 5.9 |
| Ciprofloxacin | 37196 | 2820 | 7.58 |
| Cefoperazone | 2 | 2 | 100 |
| Cefoperazone/ Sulbactum | 431 | 9 | 2.09 |
| Cefoxitin | 133 | 39 | 29.32 |
| Ticarcillin/clauvonic acid | 2 | 1 | 50 |
| Ceftazidime/clauvonic acid | 2 | 0 | 0 |
| Cefpodoxime | 2 | 2 | 100 |
| Cefotaxime | 1185 | 310 | 26.16 |
| Ceftriaxone | 38386 | 410 | 1.07 |
| Cefuroxime | 1228 | 148 | 12.05 |
| Cefazolin | 69 | 52 | 75.36 |
| Cefixime | 36046 | 446 | 1.24 |
| Ceftazidime | 1693 | 108 | 6.38 |
| Cefepime | 1110 | 67 | 6.04 |
| Cefepime/Tazobactum | 225 | 92 | 40.89 |
| Cefalexin | 91 | 25 | 27.47 |
| Chloramphenicol | 13580 | 3049 | 22.45 |
| Clindamycine | 71 | 71 | 100 |
| Co-trimoxazole | 33735 | 1323 | 3.92 |
| Colistin | 586 | 214 | 36.52 |
| Erhythromycine | 912 | 635 | 69.63 |
| Enrofloxacin | 424 | 162 | 38.21 |
| Gentamycine | 2911 | 464 | 15.94 |
| Furazolidone | 12 | 0 | 0 |
| Sulfonamide | 12 | 12 | 100 |
| Norfloxacin | 278 | 47 | 16.91 |
| Tigecycline | 204 | 53 | 25.98 |
| Cloxacillin | 15 | 1 | 6.67 |
| Oxacillin | 87 | 71 | 81.61 |
| Rifampicin | 5 | 5 | 100 |
| Kanamycine | 590 | 159 | 26.95 |
| Carbenicillin | 16 | 2 | 12.5 |
| Penicillin | 1379 | 137 | 9.93 |
| Oxytetracycline | 1302 | 698 | 53.61 |
| Sulfisoxazole | 69 | 34 | 49.28 |
| Tetracycline | 2858 | 1076 | 37.65 |
| Streptomycine | 838 | 343 | 40.93 |
| Piperacillin | 84 | 8 | 9.52 |
| Piperacillin/ tazobactum | 387 | 11 | 2.84 |
| Ertapenem | 234 | 73 | 31.2 |
| Carbapenem | 1320 | 2 | 0.15 |
| Imipenem | 1292 | 85 | 6.58 |
| Nalidixic acid | 4819 | 3578 | 74.25 |
| Tobramycine | 274 | 22 | 8.03 |
| Vancomycine | 102 | 81 | 79.41 |
| Levofloxacin | 980 | 105 | 10.71 |
| Moxifloxacillin | 225 | 79 | 35.11 |
| Ofloxacin | 1996 | 162 | 8.12 |
| Meropenem | 1065 | 114 | 10.7 |
| Nitrofurantoin | 414 | 109 | 26.33 |
| Cefotaxime + clavulanic acid | 16 | 0 | 0 |
| Ceftazidime +clavulanic acid | 16 | 0 | 0 |
| Trimethoprim | 893 | 241 | 26.99 |
| Gatifloxacin | 685 | 91 | 13.28 |
| Doxycycline | 148 | 68 | 45.95 |
| Spectinomycine | 14 | 8 | 57.14 |
| Trimethoprim+Sulphamethoxasole | 6392 | 2104 | 32.92 |
| Bacitracin | 14 | 14 | 100 |
| Sulphamethoxasole | 157 | 61 | 38.85 |
| Novobiocin | 14 | 14 | 100 |
| Clarithromycine | 234 | 104 | 44.44 |
| Moxalactum | 819 | 0 | 0 |
| Neomycine | 284 | 108 | 38.03 |
| Lincomycine | 234 | 218 | 93.16 |
| Fluroquinolone | 5500 | 3722 | 67.67 |
| Pefloxacin | 214 | 39 | 18.22 |
| Aminoglycoside | 1198 | 3 | 0.25 |
| Cefuroxime axetil | 82 | 17 | 20.73 |
| Macrolide | 1198 | 10 | 0.83 |
| Tetracycline+Azythromycine | 110 | 2 | 1.82 |
| Tera+erythro+amoxy | 110 | 19 | 17.27 |
| Tetra+erythro+azythro | 110 | 30 | 27.27 |
| Tera+erythro+cipro | 110 | 8 | 7.27 |
| Erythro+amoxy+genta | 110 | 5 | 4.55 |
| Tetra+erythro+amoxy+azythro | 110 | 14 | 12.73 |
| Tetra+amoxy+genta+nor | 110 | 5 | 4.55 |
| Ampi+cipro+nor+strepto | 110 | 6 | 5.45 |
| Ampi+chloram+cotrixa | 72 | 10 | 13.89 |

**Supplementary Table 2** Percentage of types of samples were collected in these study.

| Source | **Percentage of types of samples** | | | |
| --- | --- | --- | --- | --- |
| Animal | **Blood** | **Feces** | **Gut** | **Anal swab** |
|  | 25 | 28.57143 | 25 | 21.42857143 |
| Environment | **Food** | **Insect** | **Soil** | **Water** |
|  | 26.08696 | 30.43478 | 21.73913 | 21.73913043 |
| Human | **Blood** | **Serum** | **Stool** | **Hand swab** |
|  | 49.18033 | 11.47541 | 31.14754 | 8.196721311 |

**References**

1. Abdus Sobur M, Al Momen Sabuj A, Sarker R, et al (2019) Antibiotic-resistant Escherichia coli and Salmonella spp. Associated with dairy cattle and farm environment having public health significance. Vet World 12:984–993. https://doi.org/10.14202/VETWORLD.2019.984-993
2. Ahmed MM, Rahman MM, Mahbub KR, Wahiduzzaman M (2011) Characterization of Antibiotic Resistant *Salmonella spp* Isolated from Chicken Eggs of Dhaka City. Journal of Scientific Research 3:191–191. https://doi.org/10.3329/JSR.V3I1.6109
3. Ahmed S, Siddique MA, Rahman M, et al (2019) A study on the prevalence of heavy metals, pesticides, and microbial contaminants and antibiotics resistance pathogens in raw salad vegetables sold in Dhaka, Bangladesh. Heliyon 5:. https://doi.org/10.1016/j.heliyon.2019.e01205
4. Akhtar F, Hussain I, Khan A, Rahman SU (2010) Prevalence and antibiogram studies of Salmonella enteritidis isolated from human and poultry sources. Pak Vet J 30:25–28
5. Akter S, Sabuj AAM, Haque ZF, et al (2020) Detection of antibiotic-resistant bacteria and their resistance genes from houseflies. Vet World 13:266–274. https://doi.org/10.14202/VETWORLD.2020.266-274
6. Al Faruq A, Hassan MM, Uddin MM, et al (2016) Prevalence and multidrug resistance pattern of Salmonella isolated from resident wild birds of Bangladesh. Int J One Health 2:35–41. https://doi.org/10.14202/IJOH.2016.35-41
7. Al-Salauddin AS, Hossain MF, Dutta A, et al (2015) Isolation, identification, and antibiogram studies of Salmonella species and Escherichia coli from boiler meat in some selected areas of Bangladesh. Int J Basic Clin Pharmacol 4:999–1003. https://doi.org/10.18203/2319-2003.IJBCP20150881
8. Aslam A, Kharal SA, Aslam M, et al (2021) Trends of Antimicrobial Resistance in Typhoidal Strains of Salmonella in a Tertiary Care Hospital in Pakistan. Cureus 13:. https://doi.org/10.7759/CUREUS.12664
9. Balaji C, Balakrishnan S (2011) Screening, phylogenetic analysis and antibiotic sensitivity pattern of Salmonella enterica serovar Typhi isolates from typhoid asymptomatic carriers. Asian Pac J Trop Med 4:769–772. https://doi.org/10.1016/S1995-7645(11)60191-0
10. Ballal M, Devadas SM, Shetty V, et al (2016) Emergence and serovar profiling of non-typhoidal Salmonellae (NTS) isolated from gastroenteritis cases–A study from South India. 48:847–851. https://doi.org/10.3109/23744235.2016.1169553
11. Bandyopadhyay R, Balaji V, Yadav B, et al (2018) Effectiveness of treatment regimens for Typhoid fever in the nalidixic acid-resistant S. typhi (NARST) era in South India. https://doi.org/101177/0049475518758884 48:182–188.
12. Begum K, Reza T, Haque M, et al (2010) Isolation, identification and antibiotic resistance pattern of Salmonella spp. from chicken eggs, intestines and environmental samples. Bangladesh Pharmaceutical Journal 13:23-27
13. Dash D, Das P, Bhargava A, et al (2019) Estimating the burden of enteric fever in Chhattisgarh: A single-center study on culture-positive cases from a newly built tertiary care hospital. J Lab Physicians 11:234–239. https://doi.org/10.4103/JLP.JLP_154_18
14. Debnath T, Bhowmik S, Islam T, Chowdhury MMH (2018) Presence of Multidrug-Resistant Bacteria on Mobile Phones of Healthcare Workers Accelerates the Spread of Nosocomial Infection and Regarded as a Threat to Public Health in Bangladesh. J Microsc Ultrastruct 6:165. https://doi.org/10.4103/JMAU.JMAU_30_18
15. Dutta A, Kabir SML, Hossain MT (2018) Occurrence of Salmonella and Vibrio species in fresh fishes collected from different markets of Mymensingh, Gazipur and Sherpur districts of Bangladesh and their characterization. Asian-Australasian Journal of Bioscience and Biotechnology 3:45–51. https://doi.org/10.3329/AAJBB.V3I1.64752
16. Dutta S, Das S, Mitra U, et al (2014) Antimicrobial Resistance, Virulence Profiles and Molecular Subtypes of Salmonella enterica Serovars Typhi and Paratyphi A Blood Isolates from Kolkata, India during 2009-2013. PLoS One 9:e101347. https://doi.org/10.1371/JOURNAL.PONE.0101347
17. Fowler PD, Sharma S, Pant DK, et al (2021) Antimicrobial-resistant non-typhoidal Salmonella enterica prevalence among poultry farms and slaughterhouses in Chitwan, Nepal. Vet World 14:437–445. https://doi.org/10.14202/VETWORLD.2021.437-445
18. Garbern SC, Chu TC, Gainey M, et al (2021) Multidrug-resistant enteric pathogens in older children and adults with diarrhea in Bangladesh: epidemiology and risk factors. Trop Med Health 49:1–11. https://doi.org/10.1186/S41182-021-00327-X/TABLES/5
19. Geetha VK, Yugendran T, Srinivasan R, Harish BN (2014) Plasmid-mediated quinolone resistance in typhoidal Salmonellae: A preliminary report from South India. Indian J Med Microbiol 32:31–34. https://doi.org/10.4103/0255-0857.124292
20. Gupta S, Panday R, Sah J, et al (2020) Prevalence of Salmonella species with nalidixic acid resistance in the enteric fever patients of Nepal. British Journal of Medical & Health Sciences 2:314–321
21. Haq I, Durrani AZ, Khan MS, et al (2017) Study of antimicrobial resistance and physiological biomarkers with special reference to Salmonellosis in diarrheic foals in Punjab, Pakistan. Acta Trop 176:144–149. https://doi.org/10.1016/J.ACTATROPICA.2017.08.003
22. Hassan M, Ahaduzzaman M, Alam M, et al (2015) Antimicrobial Resistance Pattern against E. coli and Salmonella spp. in Environmental Effluents. International Journal of Natural Sciences 5:52–58. https://doi.org/10.3329/IJNS.V5I2.28612
23. Hassan MM, Begum S, Al Faruq A, et al (2018) Multidrug Resistant Salmonella Isolated from Street Foods in Chittagong, Bangladesh. Microbiol Res J Int 26:1–8. https://doi.org/10.9734/MRJI/2018/V26I630083
24. Hosain MS, Islam MA, Khatun MM, Dey RK (2012) Prevalence and Antibiogram Profiles of Salmonella Isolated from Pigeons in Mymensingh, Bangladesh. Microbes and Health 1:54–57. https://doi.org/10.3329/MH.V1I2.14090
25. Hussain A, Satti L, Hanif F, et al (2019) Typhoidal Salmonella strains in Pakistan: an impending threat of extensively drug-resistant Salmonella Typhi. European Journal of Clinical Microbiology and Infectious Diseases 38:2145–2149. https://doi.org/10.1007/S10096-019-03658-0/TABLES/3
26. Islam M, Sabrin MS, Kabir MH Bin, et al (2018) Prevalence of multidrug resistant (MDR) food-borne pathogens in raw chicken meat in Dhaka city, Bangladesh: an increasing food safety concern. Asian-Australasian Journal of Bioscience and Biotechnology 3:17–27. https://doi.org/10.3329/AAJBB.V3I1.64747
27. Islam MdK, Kabir SML, Haque AKMZ, et al (2018) Molecular detection and characterization of Escherichia coli, Salmonella spp. and Campylobacter spp. isolated from broiler meat in Jamalpur, Tangail, Netrokona and Kishoreganj districts of Bangladesh. Afr J Microbiol Res 12:761–770. https://doi.org/10.5897/AJMR2018.8945
28. Iyer RN, Jangam RR, Jacinth A, et al (2017) Prevalence and trends in the antimicrobial susceptibility pattern of Salmonella enterica serovars Typhi and Paratyphi A among children in a pediatric tertiary care hospital in South India over a period of ten years: a retrospective study. European Journal of Clinical Microbiology and Infectious Diseases 36:2399–2404. https://doi.org/10.1007/S10096-017-3073-X/TABLES/6
29. Kalambhe DG, Zade NN, Chaudhari SP, et al (2016) Isolation, antibiogram and pathogenicity of Salmonella spp. recovered from slaughtered food animals in Nagpur region of Central India. Vet World 9:176. https://doi.org/10.14202/VETWORLD.2016.176-181
30. Kansakar P, Baral P, Malla S, Ghimire GR (2011) Antimicrobial susceptibilities of enteric bacterial pathogens isolated in Kathmandu, Nepal, during 2002-2004. The Journal of Infection in Developing Countries 5:163–168. https://doi.org/10.3855/jidc.1016
31. Karim SJI, Islam M, Sikder T, et al (2020) Multidrug-resistant Escherichia coli and Salmonella spp. Isolated from pigeons. Vet World 13:2156–2165. https://doi.org/10.14202/VETWORLD.2020.2156-2165
32. Khadka P, Thapaliya J, Thapa S (2021) Susceptibility pattern of Salmonella enterica against commonly prescribed antibiotics, to febrile-pediatric cases, in low-income countries. BMC Pediatr 21:1–8. https://doi.org/10.1186/S12887-021-02497-3/FIGURES/2
33. Khan MNK, Das MR, Sabur MA, et al (2021) Isolation, identification, molecular detection and sensitivity to antibiotics of Salmonella from cattle faeces. Bulg. J. Vet. Med 24:57–66. https://doi.org/10.15547/bjvm.2019-0061
34. Khatun H, Islam S Bin, Naila NN, et al (2018) Clinical profile, antibiotic susceptibility pattern of bacterial isolates and factors associated with complications in culture-proven typhoid patients admitted to an urban hospital in Bangladesh. Tropical Medicine & International Health 23:359–366. https://doi.org/10.1111/TMI.13037
35. Koondhar MN, Kamboh AA, Khan MA, et al (2021) Antimicrobial resistance profile of Salmonella spp. isolated from raw beef meat samples collected from Karachi, Pakistan. Pak J Zool 53:2117–2122. https://doi.org/10.17582/JOURNAL.PJZ/20190929130949
36. Lamichhane B, Thakur C, Res SJ-AJPC, 2014 undefined Antibiotic resistance patterns of Gram-negative isolates in a tertiary care hospital of Nepal. Asian J Pharm Clin Res 7(3):30-33
37. Laxman Bahadur D, Ishwari Prasad D, Saroj Kumar Y, et al (2016) Prevalence and antibiotic resistance profile of *Salmonella* from livestock and poultry raw meat, Nepal. International Journal of Molecular Veterinary Research 6(1): 1-22. https://doi.org/10.5376/IJMVR.2016.06.0001
38. Mahapatra A, Patro S, Choudhury S, et al (2016) Emerging enteric fever due to switching biotype of *Salmonella (paratyphi* A) in Eastern Odisha. Indian J Pathol Microbiol 59:327. https://doi.org/10.4103/0377-4929.188124
39. Maharjan A, Dhungel B, Bastola A, et al (2021) Antimicrobial Susceptibility Pattern of Salmonella spp. Isolated from Enteric Fever Patients in Nepal. Infectious Disease Reports 2021, Vol 13, Pages 388-400 13:388–400. https://doi.org/10.3390/IDR13020037
40. Mahindroo J, Thanh DP, Nguyen TNT, et al (2019) Endemic fluoroquinolone-resistant Salmonella enterica serovar Kentucky ST198 in northern India. Microb Genom 5:e000275. https://doi.org/10.1099/MGEN.0.000275/CITE/REFWORKS
41. Mahmud MS, Bari ML, Hossain MA (2011) Prevalence of Salmonella Serovars and Antimicrobial Resistance Profiles in Poultry of Savar Area, Bangladesh. Foodborne Pathogens and Disease 8:1111–1118. https://doi.org/10.1089/FPD.2011.0917
42. Mahmud T, Hassan MM, Alam M, et al (2016) Prevalence and multidrug-resistant pattern of Salmonella from the eggs and egg-storing trays of retail markets of Bangladesh. Int J One Health 2:7–11. https://doi.org/10.14202/IJOH.2016.7-11
43. Makkar A, Gupta S, Khan ID, et al (2019) Epidemiological Profile and Antimicrobial Resistance Pattern of Enteric Fever in a Tertiary Care Hospital of North India – a Seven Year Ambispective Study. Acta Medica (Hradec Kralove, Czech Republic) 61:125–130. https://doi.org/10.14712/18059694.2018.130
44. Marjan S, Das KK, Munshi SK, Noor R (2014) Drug-resistant bacterial pathogens in milk and some milk products. Nutr Food Sci 44:241–248. https://doi.org/10.1108/NFS-05-2013-0061/FULL/PDF
45. Mazedul Haque M, Atiqur Rahman Sarker M, Rifa RA, et al (2017) Detection of food-borne bacteria in ready to eat betel leaf sold at local markets in Mymensingh. Vet World 10:1040–1045. https://doi.org/10.14202/VETWORLD.2017.1040-1045
46. Menezes GA, Harish BN, Khan MA, et al (2016) Antimicrobial resistance trends in blood culture positive Salmonella Paratyphi A isolates from Pondicherry, India. Indian J Med Microbiol 34:222–227. https://doi.org/10.4103/0255-0857.180352
47. Milton AAP, Agarwal RK, Priya GB, et al (2018) Occurrence, antimicrobial susceptibility patterns and genotypic relatedness of Salmonella spp. isolates from captive wildlife, their caretakers, feed and water in India. Epidemiol Infect 146:1543–1549. https://doi.org/10.1017/S0950268818001553
48. Misra R, Prasad KN, Amrin N, et al (2015) Absence of multidrug resistance in Salmonella enterica serotypes Typhi and Paratyphi A isolates with intermediate susceptibility to ciprofloxacin. Trans R Soc Trop Med Hyg 109:538–540. https://doi.org/10.1093/TRSTMH/TRV036
49. Mridha D, Uddin MN, Alam B, et al (2020) Identification and characterization of Salmonella spp. From samples of broiler farms in selected districts of Bangladesh. Vet World 13:275–283. https://doi.org/10.14202/VETWORLD.2020.275-283
50. Naik VK, Shakya S, Patyal A, et al (2015) Isolation and molecular characterization of Salmonella spp. from chevon and chicken meat collected from different districts of Chhattisgarh, India. Vet World 8:702–706. https://doi.org/10.14202/VETWORLD.2015.702-706
51. Narain U, Gupta R, Ganesh R, et al (2015) Emergence of resistance in community-acquired enteric fever. Indian Pediatr 52:709–709. https://doi.org/10.1007/S13312-015-0704-0
52. Naurin S, Islam MA, Khatun MM (2012) Prevalence of Salmonella in Apparently Healthy Chickens in Mymensingh, Bangladesh. Microbes and Health 1:30–33. https://doi.org/10.3329/MH.V1I1.13711
53. Nelson A, Manandhar S, Ruzante J, et al (2020) Antimicrobial drug resistant non-typhoidal Salmonella enterica in commercial poultry value chain in Chitwan, Nepal. One Health Outlook 2020 2:1 2:1–8. https://doi.org/10.1186/S42522-020-00025-4
54. Patel A, Jeyasekaran G, Jeyashakila R, et al (2020) Prevalence of antibiotic resistant Salmonella spp. strains in shrimp farm source waters of Nagapattinam region in South India. Mar Pollut Bull 155:111171. https://doi.org/10.1016/J.MARPOLBUL.2020.111171
55. Paul P, Akther S, Ali M, et al Isolation, Identification and Antibiogram Study of Salmonella sp. from Poultry Farm Environment. Int J Anim Biol. 3:5-11
56. Penjor K, Gurung M, Islam K, et al (2023) Prevalence and Antibiotic Resistance Profile of Salmonella Spp. in Broiler Carcasses from Dominant Poultry Production Areas in Bhutan. Journal of Infectious Diseases & Microbiology 1:12–24. https://doi.org/10.37191/Mapsci-JIDM-1(3)-013
57. Pokharel P, Lekhak B, Amatya R, et al (2016) Enteric fever caused by Salmonella enterica serovar paratyphi A: An emerging health concern in Nepal. Afr J Microbiol Res 10:1784–1791. https://doi.org/10.5897/AJMR2016.8281
58. Qamar A, Ismail T, Akhtar S (2020) Prevalence and antibiotic resistance of Salmonella spp. In South Punjab-Pakistan. PLoS One 15(11): e0232382. https://doi.org/10.1371/journal.pone.0232382
59. Qamar FN, Azmatullah A, Kazi AM, et al (2014) A three-year review of antimicrobial resistance of Salmonella enterica serovars Typhi and Paratyphi A in Pakistan. The Journal of Infection in Developing Countries 8:981–986. https://doi.org/10.3855/jidc.3817
60. Rahman BA, Wasfy MO, Maksoud MA, et al (2014) Multi-drug resistance and reduced susceptibility to ciprofloxacin among Salmonella enterica serovar Typhi isolates from the Middle East and Central Asia. New Microbes New Infect 2:88–92. https://doi.org/10.1002/NMI2.46
61. Rahman MA, Rahman AKMA, Islam MA, Alam MM (2018) Detection of Multi–Drug Resistant Salmonella from Milk and Meat In Bangladesh. Bangladesh Journal of Veterinary Medicine 16:115–120. https://doi.org/10.3329/BJVM.V16I1.37388
62. Rahman MM, Rahman MM, Meher MM, et al (2016) Isolation and antibiogram of Salmonella spp. from duck and pigeon in Dinajpur, Bangladesh. J Adv Vet Anim Res 3:386–391. https://doi.org/10.5455/JAVAR.2016.C177
63. Rathaur VK, Pathania M, Jayara A, Yadav N (2014) Clinical Study of Acute Childhood Diarrhoea Caused by Bacterial Enteropathogens. J Clin Diagn Res 8:PC01. https://doi.org/10.7860/JCDR/2014/6677.4319
64. Rose W, Veeraraghavan B, Pragasam AK umari, Verghese VP hilip (2014) Antimicrobial susceptibility profile of isolates from pediatric blood stream infections. Indian Pediatr 51:752–753
65. Saeed N, Usman M, Khan EA (2019) An Overview of Extensively Drug-resistant Salmonella Typhi from a Tertiary Care Hospital in Pakistan. Cureus 11:. https://doi.org/10.7759/CUREUS.5663
66. Saifullah MK, Mamun MM, Rubayet RM, et al (2016) Molecular detection of Salmonella spp. isolated from apparently healthy pigeon in Mymensingh, Bangladesh and their antibiotic resistance pattern. J Adv Vet Anim Res 3:51–55. https://doi.org/10.5455/JAVAR.2016.C131
67. Sarker BR, Ghosh S, Chowdhury S, et al (2021) Prevalence and antimicrobial susceptibility profiles of non-typhoidal Salmonella isolated from chickens in Rajshahi, Bangladesh. Vet Med Sci 7:820–830. https://doi.org/10.1002/VMS3.440
68. Saud B, Paudel G, Khichaju S, et al (2019) Multidrug-resistant bacteria from raw meat of buffalo and chicken, Nepal. Vet Med Int 2019:. https://doi.org/10.1155/2019/7960268
69. Seel SK, Kabir SML, Islam MA (2016) Molecular Detection and Characterization of Salmonella Spp. Isolated from Fresh Fishes Sold in Selected Upazila Markets of Bangladesh. Bangladesh Journal of Veterinary Medicine 14:283–287. https://doi.org/10.3329/BJVM.V14I2.31410
70. Shahunja KM, Ahmed T, Hossain MI, et al (2020) Clinical and laboratory characteristics of children under five hospitalized with diarrhea and bacteremia. PLoS One 15:e0243128. https://doi.org/10.1371/JOURNAL.PONE.0243128
71. Shahunja KM, Leung DT, Ahmed T, et al (2015) Factors Associated with Non-typhoidal Salmonella Bacteremia versus Typhoidal Salmonella Bacteremia in Patients Presenting for Care in an Urban Diarrheal Disease Hospital in Bangladesh. PLoS Negl Trop Dis 9:e0004066. https://doi.org/10.1371/JOURNAL.PNTD.0004066
72. Sharma NC, Kumar D, Sarkar A, et al (2020) Prevalence of Multidrug Resistant Salmonellae with Increasing Frequency of Salmonella enterica Serovars Kentucky and Virchow among Hospitalized Diarrheal Cases in and around Delhi, India. Jpn J Infect Dis 73:119–123. https://doi.org/10.7883/YOKEN.JJID.2019.063
73. Sharma P, Dahiya S, Manral N, et al (2018) Changing Trends of Culture-positive Typhoid Fever and Antimicrobial Susceptibility in a Tertiary Care North Indian Hospital over the Last Decade. Indian J Med Microbiol 36:70–76. https://doi.org/10.4103/IJMM.IJMM_17_412
74. Sharma P, Kumari B, Dahiya S, et al (2019) Azithromycin resistance mechanisms in typhoidal salmonellae in India: A 25 years analysis. Indian J Med Res 149:404. https://doi.org/10.4103/IJMR.IJMR_1302_17
75. Shrestha KL, Pant ND, Bhandari R, et al (2016) Re-emergence of the susceptibility of the Salmonella spp. isolated from blood samples to conventional first line antibiotics. Antimicrob Resist Infect Control 5:1–5. https://doi.org/10.1186/S13756-016-0121-8/TABLES/3
76. Siddiqui MR, Islam QT, Rahman YU, Shena TY (2015) Multiple Skin and Brain Abscess Caused by Salmonella Typhi, First Reported Case in Bangladesh. Bangladesh Journal of Medicine 26:83–85. https://doi.org/10.3329/BJMED.V26I2.25454
77. Singh BR, Singh V, Ebibeni N, Singh RK (2013) Antimicrobial and herbal drug resistance in enteric bacteria isolated from faecal droppings of common house lizard/gecko (Hemidactylus frenatus). Int J Microbiol 2013:. https://doi.org/10.1155/2013/340848
78. Singla N, Bansal N, Gupta V, Chander J (2013) Outbreak of Salmonella Typhi enteric fever in sub-urban area of North India: A public health perspective. Asian Pac J Trop Med 6:167–168. https://doi.org/10.1016/S1995-7645(13)60017-6
79. Sobur A, Hasan M, Haque E, et al (2019) Molecular Detection and Antibiotyping of Multidrug-Resistant Salmonella Isolated from Houseflies in a Fish Market. Pathogens 2019, Vol 8, Page 191 8:191. https://doi.org/10.3390/PATHOGENS8040191
80. Sohan Rodney Bangera, Umakanth S, Chowdhury G, et al (2019) Poultry: A receptacle for non-typhoidal Salmonellae and antimicrobial resistance. Iran J Microbiol 11:31–38. https://doi.org/10.18502/IJM.V11I1.702
81. Soomro AH, Khaskheli M, Bhutto MB, et al (2010) Prevalence and antimicrobial resistance of Salmonella serovars isolated from poultry meat in Hyderabad, Pakistan. Turk J Vet Anim Sci 34:455–460. https://doi.org/10.3906/vet-0908-57
82. Sugandhi P, Arvind Prasanth D (2014) Microbiological profile of bacterial pathogens from diabetic foot infections in tertiary care hospitals, Salem. Diabetes & Metabolic Syndrome: Clinical Research & Reviews 8:129–132. https://doi.org/10.1016/J.DSX.2014.07.004
83. Tegner C, Sunil-Chandra NP, Wijesooriya WRPLI, et al (2019) Detection, Identification, and Antimicrobial Susceptibility of Campylobacter spp. and Salmonella spp. from Free-ranging Nonhuman Primates in Sri Lanka. J Wildl Dis 55:879–884
84. Thamizhmani R, Bhattacharya D, Sayi DS, et al (2012) Emergence of fluoroquinolone resistance in Salmonella enterica serovar Typhi in Andaman and Nicobar Islands, India. Indian J Med Res 136:98–101
85. Uddin MB, Hossain SB, Hasan M, et al (2021) Multidrug Antimicrobial Resistance and Molecular Detection of mcr-1 Gene in Salmonella Species Isolated from Chicken. Animals 2021, Vol 11, Page 206 11:206. https://doi.org/10.3390/ANI11010206
86. Uppal B, Perween N, Aggarwal P, Kumar SK (2015) A comparative study of bacterial and parasitic intestinal infections in India. J Clin Diagn Res 9:DC01–DC04. https://doi.org/10.7860/JCDR/2015/11965.5619
87. Wajid M, Awan AB, Saleemi MK, et al (2019) Multiple Drug Resistance and Virulence Profiling of Salmonella enterica Serovars Typhimurium and Enteritidis from Poultry Farms of Faisalabad, Pakistan. https://home.liebertpub.com/mdr 25:133–142. <https://doi.org/10.1089/MDR.2018.0121>
88. Wani SA, Hussain I, Beg SA, et al (2013) Diarrhoeagenic Escherichia coli and salmonellae in calves and lambs in Kashmir: absence, prevalence and antibiogram. OIE Revue Scientifique et Technique 32:833–840. https://doi.org/10.20506/RST.32.2.2213
89. Yangzom T, Tsering DC, Kar S, Kapil J (2020) Antimicrobial Susceptibility Trends among Pathogens Isolated from Blood: A 6-Year Retrospective Study from a Tertiary Care Hospital in East Sikkim, India. J Lab Physicians 12:03–09. https://doi.org/10.1055/S-0040-1712814/ID/JR_20/BIB
90. Yasmin S, Nawaz M, Anjum AA, et al (2020) Antibiotic susceptibility pattern of Salmonellae isolated from poultry from different districts of Punjab, Pakistan. Pak Vet J 40:98–102. <https://doi.org/10.29261/PAKVETJ/2019.080>
