# Supplementary figures and images for: Prevalence and Antimicrobial Resistance Profile of Salmonella Isolated from Human, Animal and Environment Samples in South Asia: A 10-Year Meta-analysis
Source: J Epidemiol Glob Health. 2023 Oct 26;13(4):637–52. doi: 10.1007/s44197-023-00160-x (PMC10686918; doi:10.1007/s44197-023-00160-x)

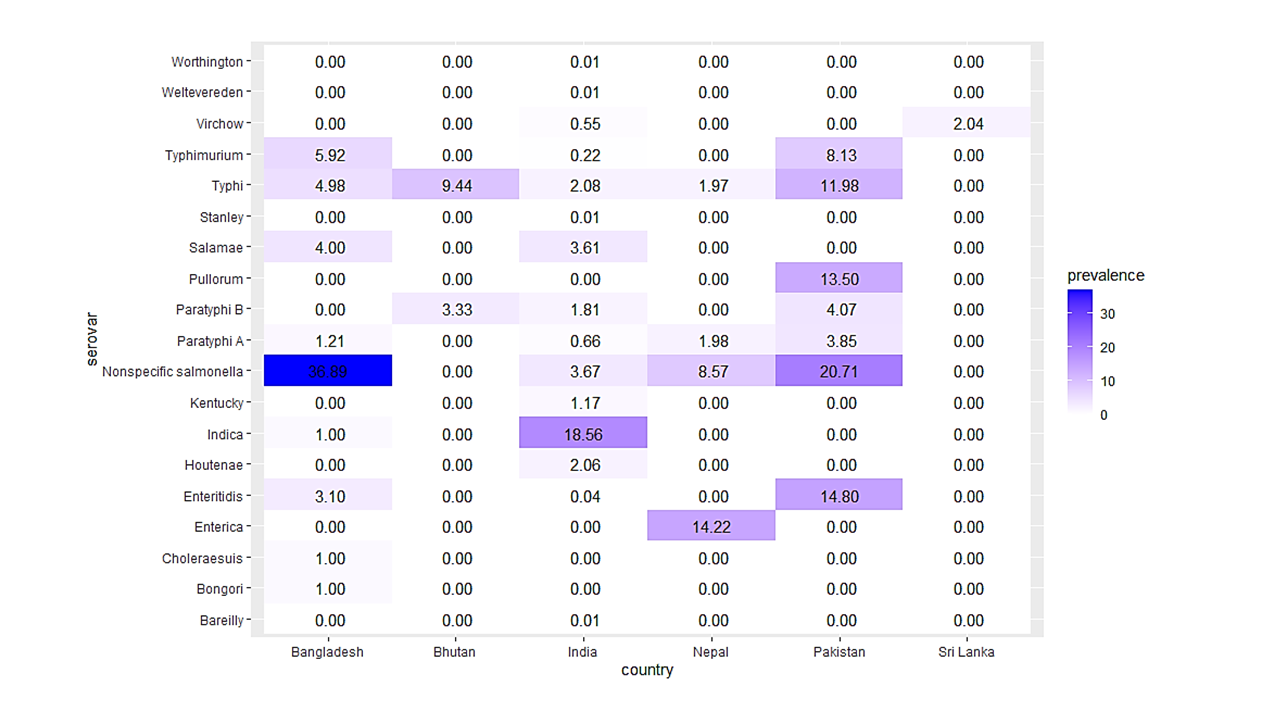
**Supplementary Fig. 1** Prevalence of different *Salmonella* serovar in different countries

Supplement: Supplementary file 4 — Supplementary file4 (DOCX 374 KB) [file 44197_2023_160_MOESM4_ESM.docx]
